# Diagnostic challenge in children with an acquired demyelinating syndrome: an illustrative case report

**DOI:** 10.3389/fnins.2023.1205065

**Published:** 2023-07-20

**Authors:** Luciana Midaglia, Ana Felipe-Rucián, Ignacio Delgado Alvarez, Xavier Montalban, Mar Tintoré

**Affiliations:** ^1^Servei de Neurologia/Neuroimmunologia, Multiple Sclerosis Centre of Catalonia (Cemcat), Hospital Universitari Vall d'Hebron, Universitat Autònoma de Barcelona, Barcelona, Spain; ^2^Secció de Neurologia Pediàtrica, Hospital Universitari Vall d'Hebron, Universitat Autònoma de Barcelona, Barcelona, Spain; ^3^Servei de Radiología, Hospital Universitari Vall d'Hebron, Universitat Autònoma de Barcelona, Barcelona, Spain

**Keywords:** acquired demyelinating syndrome (ADS), myelin oligodendrocyte glycoprotein (MOG), MOG antibody-associated disease (MOGAD), neuromyelitis optica spectrum disorder (NMOSD), multiple sclerosis (MS), pediatric onset multiple sclerosis (POMS)

## Abstract

The clinical-radiological and biological overlap of the spectrum of pediatric demyelinating disorders makes the diagnostic process of a child with an acquired demyelinating syndrome truly challenging. We present a 9-year-old girl with subacute symptoms of severe decrease in bilateral visual acuity and gait ataxia. An urgent MRI showed inflammatory-demyelinating lesions affecting the periaqueductal gray matter, the cerebellar hemispheres, the area postrema as well as both optic nerves and chiasm. Likewise, multisegmental involvement of the cervical and dorsal spinal cord was found, with short and peripheral lesions. Anti myelin oligodendrocyte glycoprotein (MOG) antibodies (Abs) were positive in cerebrospinal fluid (CSF) and weakly in serum. Oligoclonal bands (OB) were positive in CSF. Based on all this, the diagnosis of MOG antibody disease (MOGAD) with a neuromyelitis optica spectrum disorder (NMOSD)-like picture was made. Given the good clinical and radiological recovery after the acute phase treatment, and that anti MOG Abs became negative, it was decided to keep the patient without specific treatment. However, during follow-up, while the patient was asymptomatic, a control brain MRI showed the appearance of new lesions with morphology and topography suggestive of multiple sclerosis (MS). This, added to the presence of OB, made the diagnosis of pediatric-onset MS (POMS) likely. Immunosuppressive treatment was restarted with a good response since then. Unlike adult-onset MS, children with POMS may usually not have entirely typical clinical and radiological features at presentation. In many cases, the time factor and close clinical and radiological monitoring could be critical to make an accurate diagnosis.

## Introduction

Acquired demyelinating syndrome (ADS) is defined as the first event of neurological dysfunction, acute or subacute onset, associated with evidence of inflammatory demyelination of the central nervous system (CNS), including the optic nerves (Absoud et al., 2013).

The first demyelinating event or ADS in children ≤18 years can have a very varied clinical and radiological expression. The main diagnoses that encompass an ADS in the pediatric age are acute disseminated encephalomyelitis (ADEM), multiple sclerosis (MS), neuromyelitis optica spectrum disorder (NMOSD), and myelin oligodendrocyte glycoprotein (MOG) antibody disease (MOGAD) (Luchesa Smith et al., 2022).

Although important advances have occurred in recent years in terms of establishment of refined diagnostic criteria, identification of anti MOG and aquaporin-4 (AQP4) antibodies (Abs), as well as progress in therapeutic management, the clinical-radiological and biological overlap of the spectrum of pediatric demyelinating disorders, makes the diagnostic process of a child with ADS truly challenging (Krupp et al., 2013; Chitnis, 2019).

In this context, we will describe an illustrative case report.

## Case report

**Figure 1:**
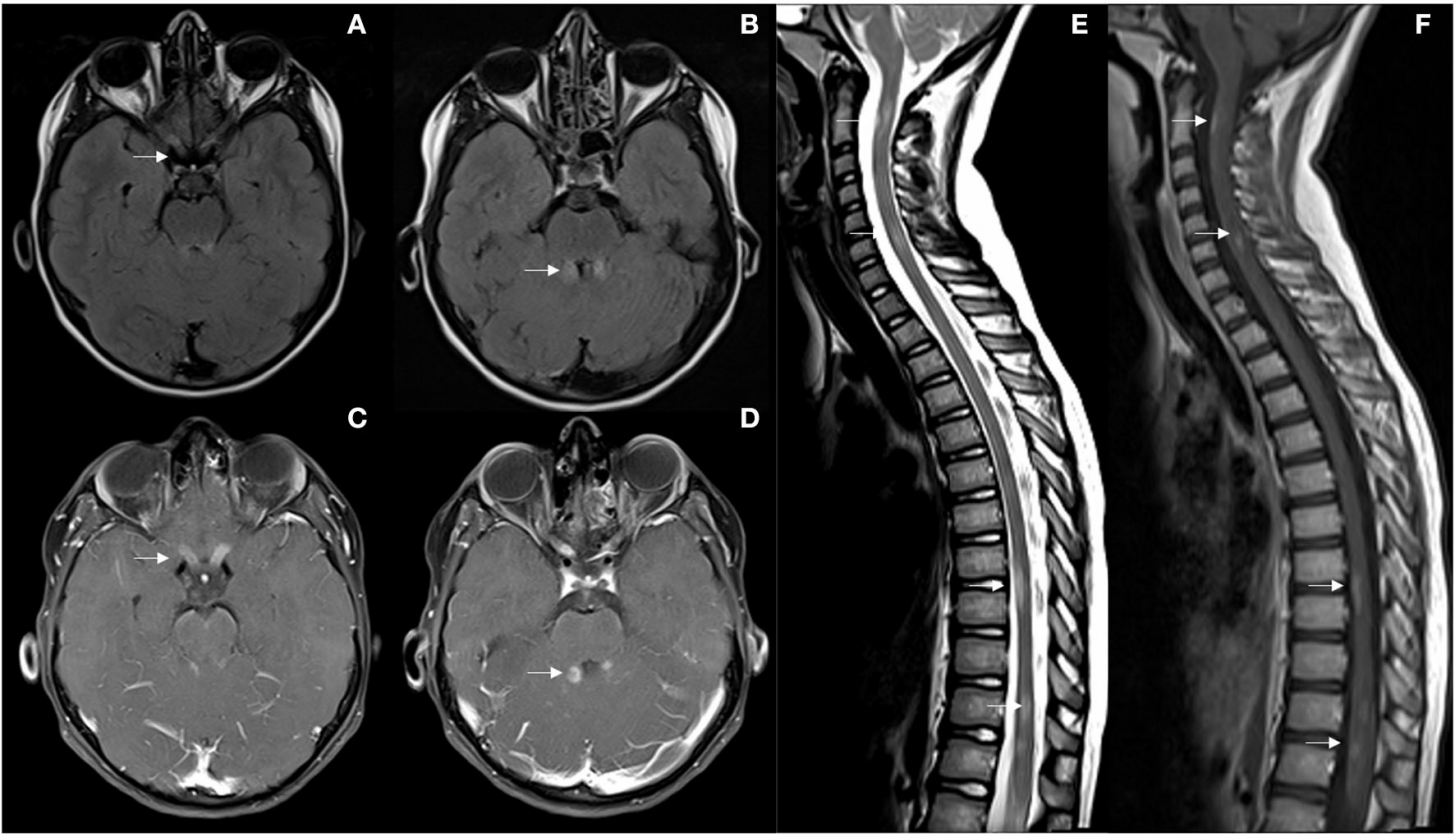
**(A, B)** First brain MRI showing optic nerves, superior cerebral peduncles and periaqueductal FLAIR hyperintensities (arrows). **(C, D)** Ill-defined enhancement after gadolinium administration, and uptake along both optic nerves. **(E, F)** Multisegmental peripheral involvement of the spinal cord with, C2, C6, D8-D9, and D11-D12 lesions with ill-defined uptake after gadolinium (arrows).

We present a 9-year-old girl, from Morocco, with no medical history except for type 1 diabetes mellitus in her father. In December 2019, the patient was referred to the Emergency Department of our hospital due to decreased visual acuity in both eyes for 3 days, associating ocular pain with eyes mobilization. In the last 24 h she added gait disturbance and vomiting. The week before symptoms onset, the patient had presented an upper respiratory infection with fever. The first ophthalmological assessment revealed a visual acuity (VA) of 0.05 in the right eye (RE) and 0.08 in the left eye (LE) with raised and poorly defined papillae, predominantly in the LE. The neurological examination showed dysarthria, nystagmus, ataxic gait, generalized hyperreflexia and bilateral Babinski. Ambulation without support was not possible. The physical disability measured by the Expanded Disability Status Scale (EDSS) was 6.0. An urgent brain and spinal cord MRI was performed, which identified subtle lesions affecting the posterior fossa, specifically the superior cerebral peduncles, periaqueductal gray matter, right middle cerebellar peduncle, both cerebellar hemispheres, and the area postrema. A subtle involvement of the right thalamic nucleus and subcortical white matter of right frontal and temporal lobes was observed, with hypersignal of both optic nerves and chiasm, as well as the diencephalon. After gadolinium administration, multiple lesions involving the brainstem and cerebellum showed poorly defined cotton-wool enhancement, with very striking uptake along both optic nerves. Likewise, multisegmental involvement of the spinal cord was found, specifically at the cervical (C2 and C6) and dorsal levels (D8-D9 and D11-D12). Lesions were short and peripheral, with ill-defined uptake after gadolinium (see Figure 1). The etiological study was completed with a lumbar puncture that detected 7 cells, elevated IgG index and presence of oligoclonal bands (OB) in cerebrospinal fluid (CSF) and additional identical OB in both serum and CSF. The CSF microbiological study (viral PCRs, culture) was negative, while anti MOG antibodies (Abs) test was positive (anti AQP4 negative). The serum systemic autoimmunity panel (ANA, ENAs, ANCA) was negative, while anti MOG Abs were weakly positive 1/80 (anti AQP4 negative). The MOG Abs were detected by a cell-based assay (CBA). Visual evoked potentials showed an absence of response bilaterally, but with preserved functionality of the visual pathway to the simple stimulus (Flash). Treatment with methylprednisolone 1 g/day intravenously was started on the same day of admission completing a total of 5 days followed by maintenance oral corticosteroid therapy and a subsequent tapering regimen. Simultaneously, intravenous gamma globulins were administered at a total dose of 2 g/Kg (2 doses of 1 g/Kg every day, during 2 consecutive days). Given the persistence of severely diminished vision and gait instability (EDSS 5.5), treatment of the acute phase was reinforced with 5 daily sessions of immunoadsorption with immunoglobulins (100 mg/kg/day) during the first 5 days and subsequently 5 more sessions every 48–72 h. Finally, she received a first dose of rituximab 375 mg/m^2^ in January 2020, up to a total of 4 weekly doses. The control MRI performed 1 month after the symptoms onset, showed a clear radiological improvement of the demyelinating lesions affecting the posterior fossa and spinal cord, with subtle subependymal and left optic nerve involvement persisting. No signs of radiological activity were found after contrast administration. On discharge, despite the best treatment, the girl still suffered from a severe visual deficit (VA RE 0.15 and LE finger count at 1 meter) with improvement in other symptoms, EDSS 4.0. Fortunately, the patient showed a slow but progressive improvement in visual deficit over time, reaching a bilateral VA of 1.0 at 4-month follow-up. Serology for anti MOG Abs became negative in March 2020, 3 months after the first test, persisting negative in the controls carried out in June 2020, April 2021, May 2022 and January 2023. A new MRI performed 6 months later, confirmed the radiological improvement already observed in the previous scan. Given the good clinical and radiological recovery, and that anti MOG Abs became negative, in the context of a first and unique neurological event, it was decided to keep the patient without specific treatment.

**Figure 2:**
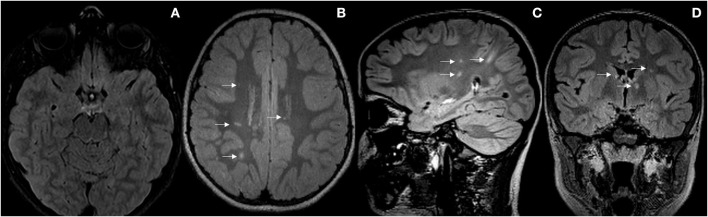
**(A)** Radiological improvement of the demyelinating lesions affecting the posterior fossa. **(B–D)** New focal lesions in the right frontoparietal subcortical, left parietal juxtacortical white matter, left internal capsule, periventricular and callous-septal (arrows).

As the patient remained asymptomatic, a control MRI scan was performed after 1 year and 3 months of clinical onset, that surprisingly showed new focal lesions in the right frontoparietal juxtacortical and subcortical white matter, right temporal periventricular, left internal capsule, left corona radiata, and callous-septal, with persistence of small lesions in the left upper cerebral peduncles, in the periaqueductal gray matter, right middle cerebellar peduncle, and right thalamic nucleus, as well as persistence of the discrete hypersignal of the left optic nerve and chiasm. In the spinal cord, bilateral posterior marginal punctate lesions persisted. None of the lesions showed enhancement after gadolinium administration (see Figure 2). At that time, the patient's case was presented to the Clinical-Radiological Committee for the diagnosis of inflammatory diseases of the CNS of our hospital. It concluded that although the initial findings were highly suggestive of MOGAD with an NMOSD-like picture, the morphology and topography of the new lesions appeared on the last MRI, added to the presence of OB in the CSF, made the diagnosis of MS the most likely. Thus, immunosuppressive treatment with rituximab was restarted at a dose of 500 mg × 2 separated by 14 days, every 6 months. Since then, the patient remains asymptomatic until the present date with adequate tolerance to treatment. Subsequent MRI scans performed 2 years, 2 years and 6 months, and 3 years from clinical presentation showed no new lesions or signs of inflammatory activity. The last neurological examination carried out at 3-year follow-up, only showed generalized hyperreflexia (EDSS 1.0).

## Discussion

We present a wellstudied case of a girl who presented ADS clinically and radiologically highly suggestive of MOGAD with an NMOSD-like picture at baseline, and low-titer positive anti MOG Abs, but with positive OBs as a red flag. During follow-up, and in the absence of new symptoms, the MRI lesions changed in morphology and topography, transforming radiologically into MS. Unlike adult-onset MS, children with pediatric-onset MS (POMS) may usually not have entirely typical clinical and radiological features at presentation (Yeh et al., 2016; Tenembaum, 2017).

Initially, in the case of our patient, the bilateral and extensive involvement of optic nerves with compromise of the optic chiasm on MRI was highly suggestive of NMOSD. This in turn was supported by the clinical association of vomiting and ataxic gait, with radiological repercussions at the periaqueductal gray matter and the area postrema. Although not the most typical findings, spinal cord involvement, with short and eccentric lesions, has also been described in NMOSD (Flanagan et al., 2015). The subsequent detection of positive anti MOG Abs in CSF and weakly positive in serum made the diagnosis of MOGAD with an NMOSD-like picture the most plausible. It is known that, although ADEM is the most usual clinical presentation in young children with positive anti MOG Abs, older patients (>9 years) are more likely to present as optic neuritis or NMOSD-like (Hacohen et al., 2018). So far, everything seemed consistent, except for the presence of OB in the CSF, which is rare (≈10%) to find in both, MOGAD and NMOSD (Fadda et al., 2021a). Concerning this red flag, while the patient was asymptomatic during follow-up, brain MRI scans adopted a typical radiological expression of MS, fulfilling the dissemination in space and time of revised 2017 McDonald criteria (Thompson et al., 2018). New MRI silent lesions are rare in MOGAD children (<15%). When detected, they may occur within the first year of follow-up, especially in the three months from presentation, and usually resolve completely over time. Unlike MS, most silent lesions in MOGAD are typically ill-defined and involve the thalamus, brainstem, and around the fourth ventricle (Waters et al., 2020; Fadda et al., 2021b; Sechi et al., 2022). Additionally, the clinical significance of anti MOG Abs in CSF is currently unclear. The synthesis of anti MOG Abs in the CSF of patients with MOGAD can be detected from the onset of the disease and can support the diagnosis of MOGAD in seronegative patients with highly suggestive symptoms. Recently, it has been associated with a more severe clinical presentation and a worse prognosis (Carta et al., 2023). However, CSF anti MOG Abs could also be present in ADS other than MOGAD, including in MS (Nam et al., 2022). A recent study identified 4 children with diagnosis of POMS with unique MOG-IgG in the CSF (Armangue et al., 2020).

Therefore, considering the complete trajectory of the patient's disease from the beginning to the present, the diagnosis of MS seems to be the most appropriate option, but so far not definitive given the short follow-up period. As occurred in our patient, and although initially atypical, optic nerve involvement in POMS, especially in children under 10 years of age, could more commonly be bilateral with severe loss of visual acuity (Absoud et al., 2011). Furthermore, MRI lesions in POMS have also been described as large and with ill-defined borders (Tenembaum, 2017). Additionally, the presence of anti MOG Abs in serum has been reported in up to 5% of pediatric patients with MS (Fadda et al., 2021a). All of this supported by the typical baseline characteristics for MS that the patient already presented, such as the presence of OB in CSF and short and peripheral lesions in the spinal cord MRI (Fadda et al., 2021a). In the absence of a completely accurate diagnosis, we opted to reintroduce and maintain the patient under treatment with rituximab, a proven effective drug in both MS and MOGAD (Midaglia et al., 2018; Whittman et al., 2020).

In this way, the true challenge that clinicians face when dealing with children presenting a first event of an inflammatory demyelinating disease has been illustrated. In many cases, the time factor and close clinical and radiological monitoring could be critical to make an accurate diagnosis and initiate/change the therapeutic approach opportunely.

## Data availability statement

The raw data supporting the conclusions of this article will be made available by the authors, without undue reservation.

## Ethics statement

Written informed consent was obtained from the individual(s) for the publication of any potentially identifiable images or data included in this article.

## Author contributions

LM, AF-R, and ID contributed to the conception, design, and writing of the manuscript. XM and MT contributed to the conception and design of the manuscript. All authors contributed to the article and approved the submitted version.
